# Barriers and facilitators of the implementation of the application of pelvic floor muscle training in patients with prostate cancer: a scoping review

**DOI:** 10.3389/fpubh.2023.1191508

**Published:** 2023-09-12

**Authors:** Lijuan Wang, Yaqin Li, Ziyi Qi, Wei Wang

**Affiliations:** Department of Nursing, The First Affiliated Hospital, Zhejiang University School of Medicine, Hangzhou, China

**Keywords:** prostate cancer, barriers, facilitators, pelvic floor muscle training, scoping review

## Abstract

**Background:**

Patients with prostate cancer (PCa) benefit significantly from pelvic floor exercises, but recent results indicate that these exercises have not been fully promoted in clinical settings. This scoping review aimed to identify the facilitators of and barriers to pelvic floor muscle training (PFMT) in PCa survivors.

**Methods:**

A scoping review was conducted in November 2022. Relevant studies were identified from CINAHL, Embase, PubMed, PsycINFO, and Web of Science databases from their inception to 20 November 2022. Data were analyzed and extracted by two formally trained researchers.

**Results:**

A total of 53 studies were included, most of which were randomized controlled trials. The Tailored Implementation for Chronic Diseases (TICD) model framework was used to identify the contents of seven barriers and promotion areas, as well as a series of sub-domains. The most common barriers to implementing pelvic floor muscle training (PFMT) included the following: the lack of a common scheme in guidelines and the measurement of common standardized outcomes, inadequate self-monitoring or feedback from healthcare professionals to improve PFMT compliance, poor patient compliance, and a lack of implementation equipment and financial support. Good treatment effects and easy operation were the facilitators of PFMT.

**Conclusion:**

The implementation of PFMT faces several challenges and opportunities that should be understood thoroughly before implementation. In terms of guidelines and clinical practice, more work is needed, and the possibility of PFMT implementation in various hospitals and community health centers or clinics should be considered.

## 1. Introduction

In 2021, prostate cancer (PCa) surpassed lung cancer as the second most prevalent tumor-specific cause of death among men globally (1). In 2020, it was predicted to cause 1.4114 million new cases and 375,000 fatalities worldwide (2). The incidence rates are rising globally, particularly in Asia, Northern Europe, and Western Europe (3). Although radical prostatectomy can increase a patient’s chance of survival, it can also disrupt their body’s natural function, reduce masculinity (4), and result in poor quality of life (QoL) (5). Despite improved treatment regimes, PCa treatment has several side effects. Urinary incontinence (UI) and erectile dysfunction (ED) were the most common complications of radical prostate resection (6). UI frequently occurs after catheter removal, and it has a significantly negative impact on a patient’s function and health-related QoL, particularly in the first 6 months after surgery. Patients who have UI after radical prostatectomy are typically advised to seek conservative treatment. Common conservative treatment strategies include behavior modification and pelvic floor muscle exercises (PFMEs), lifestyle education, artificial urinary sphincter, and pharmacological treatments (7). All conservative treatment trials after radical prostatectomy show moderate evidence that pelvic floor muscle training (PFMT) has an overall benefit in reducing UI compared with control management (8).

PFMT is an affordable and uncomplicated procedure that can help with bladder control, bowel control, and sexual function, and it can reduce the likelihood of UI. To prevent an increase in intra-abdominal pressure, PFMT is provided to participants to teach them how to use biofeedback to synchronize their voluntary pelvic floor muscle contractions and time them precisely (8). Biofeedback refers to the use of specific equipment for visual and auditory feedback training during pelvic floor muscle functional exercises to strengthen the muscles involved. The theory behind PFMT is that the pelvic floor may become stronger and more effective if specific pelvic floor muscles are repeatedly and voluntarily contracted during increased intra-abdominal pressure, which will inhibit detrusor activity (9). Additionally, PFMT may cause the periurethral tissues to increase external mechanical urethral pressure (10). It is believed that repeated contractions can improve voiding control by increasing support for the detrusor and urethral sphincter muscles. PFMT includes different modalities such as simple Kegel exercises, biofeedback (verbal or machine-mediated), electrical stimulation (ES) *via* surface electrodes, and extracorporeal magnetic innervation (8). These interventions can be compared with each other—individually or in combination—and to no treatment.

The advantages of pelvic floor rehabilitation exercise after PCa diagnosis include improvements in continence in the short term (11), reductions in nocturia and decreased use of pads (12), improvements in ED results (13), and rapid improvements in QoL (14). It has been shown that exercise of the pelvic floor muscles is safe for older PCa patients with signs of advanced disease, such as bone metastases. PFMT is a critical intervention for cancer patients since it significantly reduces certain adverse effects of treatment (15). Active pelvic floor exercises help avoid UI by improving muscle strength and endurance. Guidelines (16, 17) suggest that PFMT alone or combined with biofeedback or ES is effective in treating postprostatectomy incontinence (PPI). The timing of pelvic floor muscle functional exercises for PCa includes preoperative and postoperative radical prostatectomy for PCa, and both preoperative and postoperative PFMT can improve UI (15).

Despite extensive research in recent years indicating that PFMT can help reduce UI after prostatectomy, it has not been widely promoted in a significant proportion of hospitals and communities due to human and social factors. During clinical care rehabilitation, there is a significant gap between postoperative exercises for PCa and evidence-recommended practices of guidelines. This review aimed to understand the promotion, motivations, preferences, and barriers to PTME participation among men after PCa surgery by conducting a thorough literature search.

## 2. Methods

We conducted a scoping review of the literature on PFMT among men with PCa, following the Preferred Reporting Items for Systematic Reviews and Meta-analyses (PRISMA) guidelines (18). The scoping review is a rigorous method for mapping research and presenting results in a format that is accessible to knowledge users and is an increasingly common approach to mapping broad topics. The six-step framework prescribed by Arksey and O’Malley, advanced by Levec et al. (19), includes (1) identifying the research question, (2) identifying relevant literature, (3) selecting the relevant study, (4) charting the data, and (5) collating, summarizing, and reporting the data. The review team included knowledge users and a PCa survivor involved in the consultation process. The team was formed to ensure that the findings are relevant to physical activity and exercise provision efforts and to facilitate the dissemination of findings.

### 2.1. Identifying the research question

The researchers discovered the research problem through an early literature review. Our research topic focused on the factors, preferences, and barriers to PFMT in men with PCa. PFMT, previously also known as Kegel exercises, is a behavioral training method to prevent and treat UI by strengthening the pelvic floor muscle that supports the pelvic organs through the voluntary and repetitive contraction and relaxation of the pelvic floor muscles. For the purposes of this review, the following forms of functional exercises for the pelvic floor muscles were included: PFMT alone, PFMT with biofeedback, ES with PFMT and biofeedback, and pelvic floor education.

### 2.2. Identifying relevant literature

The search strategy was developed after consulting a reference librarian who suggested using MeSH terms to find the most relevant studies and public health specialists in systematic reviews. It was developed in consultation with a health sciences librarian specialized in PFMT after PCa surgery to ensure an appropriate and thorough search of the literature. Articles were searched in PubMed, CINAHL, Embase, PsycINFO, and Web of Science databases. The timeframe of the literature search was from the database inception to 20 November 2022. The following key search terms were searched in each database: (PCa OR prostate neoplasm OR prostatic neoplasms OR prostate tumor) AND (pelvic floor muscle training OR pelvic floor muscle exercise OR pelvic floor muscle strengthening OR Kegel exercise OR PFMT/PFE/PFME). Hand searching the reference lists of included studies identified additional pertinent articles for evaluation. All included studies were limited to those published in English language.

### 2.3. Selecting the relevant study

The following principles governed the inclusion and exclusion criteria of the review. The subjects were patients undergoing radical prostatectomy. The interventions included pelvic floor muscle training to prevent UI or to improve sexual function, QoL, and other outcome indicators. The type of research articles included were original peer-reviewed publications (randomized controlled trials, cross-sectional studies, and mixed-methods studies); meta-analyses, reviews, study protocols, abstracts, posters, conference papers, fertility studies, animal studies, and editorial comments were excluded.

### 2.4. Charting of the data

Two authors (LJ and YQ) independently screened the titles of the identified references and excluded ineligible studies. One author (LJ) screened all the abstracts and ranked them as relevant, irrelevant, or unsure. The second author (YQ) double-screened 50% of the abstracts to ensure consistent application of the eligibility criteria. Two authors extracted data using standardized checklists to assess the quality of studies and evidence synthesis. A third author (ZY) arbitrated studies when the first two authors were uncertain about their eligibility. Studies ranked as irrelevant by both reviewers were excluded. The following information was extracted: first author, country, year, study design, age, sample size, the purpose of the study, intervention time, and details of intervention methods. Any differences in opinion were resolved through discussion.

### 2.5. Collating, summarizing, and reporting the data

The research team created a summary of all the facilitators and barriers in accordance with the framework of the Tailored Implementation for Chronic Diseases (TICD) (20) to identify the important practice factors (see Table 1). The concept of practice determinants is categorized into seven TICD domains, namely, guidelines factors, personal health professional factors, patient factors, professional interactions, incentives and resources, organizational change capabilities, and social, political, and legal variables. According to the research team, the framework is ideal for summarizing the application and implementation of an intervention strategy. We identified and coded promoters and obstacles using deductive and inductive topic methods to map the data (73). From the findings of each publication, one of the authors (YQ) retrieved the barriers or facilitators that were initially gathered. Thematic synthesis was used to arrange the facilitators and barriers into recurrent themes in the studies, considering the frequency of reports (Table 1) or the number of times they were designated as relevant issues in specific investigations. The quantity of replies usually determines the frequency of barriers or facilitators in quantitative studies (questionnaires). Therefore, the more prevalent subjects in the literature are viewed as the typical deterrents or promoters of PFME modifications.

**Table 1:** Barriers and facilitators from the TICD framework.

Domain	Sub-domain	Barriers	Facilitators
Guideline factors	Recommendation-clarity	Self-reported continence status is imprecise (12) to grade the incontinence of patients before and after treatment (12) the subjective method of evaluating incontinence and the wide range in interval between surgery and initiating treatment (21). It is unclear whether the theoretical basis of PFE, working well in women with stress incontinence, can be applied directly to men (22) lack of standardized treatment protocols (16) the definition of incontinence and the method used to measure incontinence is crucial (23) inconsistent definition of continence and the variation in follow-up (24) the studies included patients with differing severities of UI, including minimal or mild UI that can resolve spontaneously, and the natural rate of resolution is often not considered (25)	Early exercise after surgery is recommended (26, 27). A more objective evaluation of UI would be the urodynamic evaluation (28)
	Recommended clinical intervention-feasibility		Simple to deliver (29) the use of biofeedback or electrical stimulation does not appear to be essential makes intervention more practical (30) physical therapy is not invasive (28, 31–33) easy to perform (34, 35) can practice the pelvic floor muscle contraction exercises themselves independently at home without assistance from family members (35) painless (33) reduced PPI and improved QoL outcomes related to incontinence (36) non-harmful method to reduce the duration and the degree of PPI and improve quality of life (28)
	Recommended clinical intervention- Accessibility of the intervention	Men who cannot learn to control their pelvic floor muscles using verbal coaching (30). Inappropriate timing of intervention (8) the lack of long-term follow-up (37)	PFM training of longer duration prior to surgery, or of higher frequency and/or intensity, is more likely to be beneficial (36, 38)
	Recommended behavior-compatibility	Lacked in methodology and in homogeneity of either continence definition, or sample, or in instruments to measure the severity of the incontinence or in the workout of the rehabilitation program (27) extensive sphincter damage or severe bladder dysfunction can hardly benefit from PTFM (39)	
	Recommended behavior-observability	No significant influence on urinary incontinence (31, 34, 40–45) natural course of post-prostatectomy incontinence indicates a rapid improvement in the majority of patients even without a specific training (31). No sufficient effect of erectile dysfunction (34, 40, 43, 44) there was not reflected in better outcome in HRQoL parameters (45, 46) limited benefit in long-term benefit of PFE training	Positive effect on urinary incontinence (12, 21–23, 25, 27–30, 32, 35, 37–39, 46–64, 56;57) improvement of nocturia and decreased use of pads (12) had superior results on erectile dysfunction (26, 33, 54) positive effect on sexual dysfunction (18) LUTS intensity and distress improved (65) rapid improvements in urinary symptoms and quality of life (55–57, 61, 63, 66) prehabilitation hastens return to baseline for functional capacity and reduces preoperative and 6-month postoperative anxiety (67) reducing the waist perimeter (38) earlier achievement of urinary incontinence (68)
Individual health professional factors	Knowledge and skills-domain knowledge	Urologists’ knowledge of public sector providers of PFMT was limited (29) urologist lack of knowledge of which muscles are involved and which fibres need to be contracted (27) urologist lack of knowledge about normal postoperative events (64)	The rate and the time to continence is shortened if patients are submitted to a postoperative personal training program of pelvic muscle re-education supported by physicians and nurses experienced in continence disorders (27)
	Knowledge and skills-Awareness and familiarity with the recommendation	Objective UI indicators was needed (34) the muscle-targeted intensity of the programme and the position in which pelvic floor muscle contraction are influential factors and contribute to variation in intervention outcomes (69)	The training effect might have been greater had we used more intensive preoperative training or resumed intervention after surgery with a more regular program of postoperative visits to further optimize outcomes (62)
	Knowledge and skills-knowledge about own practice	Surgical skill is crucial influence the final outcome but unmeasurable (21, 23)	Moderate exercise intensity (49) a more intense and/or longer exercise regimen could afford better result (26, 51)
	Knowledge and skills-skills needed to adhere	Lack of surgical expertise (32, 37) the intervention might have been biased by the surgeon learning curve (39)	Appropriate comprehension of instructions and technique (65) objective documentation of ability to perform the exercises (65)
	Professional behavior-capacity to plan change	Physical therapy result may dependent on the ability to find the best individual treatment scheme (31) managed only with verbal instructions for exercises (57) the lack of evaluation of patients’ autonomy after PFMT finished (38) lack of financial comparison (68) did not assess the basic knowledge of this cohort of pelvic floor exercise prior to enrollment (68)	The initiation of the training programme soon after surgery (21) early recruitment (40) the inclusion of female partners (40). The intervention was manualized and based on a solid theoretical framework adjusted to the couples needs accordingly (40) randomized controlled trials should be well-designed, with adequate sample sizes, validated outcome measures and long-term follow-up periods (42) extended follow-up of participants (65) multicenter RCT design (67) large sample (67) the consistency and robustness of the findings (44) standardization in the delivery of the intervention (44) there are no side-effects or risks from therapy (60)
	Professional behavior-self-monitoring or feedback	Relatively small number of patients (34, 35, 38, 42, 47, 58, 59, 61) short follow-up period of time (32, 47) baseline losses are not homogeneous (49). No corresponding control group (35, 50, 54) many study subjects suffering from minor incontinence problems at baseline (32) no longitudinal period (35) must assure that training exercises are being properly performed (53) the absence of an instrument to measure the patient’s adherence to perform the exercises at home in the group that received only guidance for home exercises (43) the influence of drug factors was not considered (33) inappropriate instructions from the staff (66) the lack of assessment of long-term treatment outcomes and a rather small study group and small difference between groups at the baseline point (70) exclusive use of subjective measures (61)	Individual follow-up of the patients might have improved our continence rates (48) randomized design, with similar treatment and control groups, a strict study protocol, in which all randomized patients were analyzed, and a team of blinded evaluators (34). Providing intervention over a longer time period may prove more effective (39)
Patient factors	Patient motivation	Physical therapy result may dependent on patient motivation (31) patients who perform exercises at home cannot be controlled for adherence to the exercise program (49). Fatigue is one of the major issues of PFM dysfunction (36)	Empowers patients to take charge of their urinary health (32). The dropout rate is relatively low (33) a high adherence rate for the entire intervention (67)
	Patient behavior	Need better compliance and persistence (71) main reasons for patients to decline the intervention: the lack of transportation and time (52) declining participation because of distance and travel to the study site (56, 67) exercises should kept up on a regular basis (38)	Completion of the training programme (65)objective measures of adherence to prescribed pelvic floor exercise regimens (65) intensive and supervised programmes have produced better results than self-training programmes (66). It is critical that men be knowledgeable about self-care activities to manage the surgical side effects (72) the promotion of adherence from the start of the program onwards favored the autonomous performance of exercises at the patients’ homes (38)
Incentive and resources	Availability of necessary resources	Not one of the local public providers was called upon to provide preoperative PFMT over the post-intervention period (29) physical therapy result may dependent on the teaching tools used (31) resources for supporting continuing care or family burdens (52). A barrier determined by a series of factors relating to age, gender, and the social and demographic context could prevent access and provision of aid to older adult people in need of assistance (38) relatively high withdrawal rate (63) lack of professional healthcare support (64)	Reduces reliance on technology and hospital facilities (32)
	Continuing education system		Patient education is crucial for facilitating participation and adherence with treatment recommendations (52)
	Financial incentives and disincentives		The use of a low-intensity, supervised programme less time-consuming for caregivers, more feasible for peripheral urological clinics and probably more cost-effective (47)
Professional interactions	Team processes	Physical therapy result may dependent on the dedication of the physical therapist (31) the value of regular in-person contact with the physiotherapist have been underestimated (46) nurses or other personnel must be trained in these biofeedback equipment techniques (41). A time-consuming and therefore expensive programme of intensive guidance by a physiotherapist does not seem to be necessary (59)	Efficacy of PFMT is dependent on the interaction with a health-care professional (22) close monitoring of the patient by the physical therapist (34) Utilizing physiotherapists with a special interest in PFME is the key to the success of the present study (39). Group setting likely increases patients motivation to maintain PFME (61) patient education in pelvic floor musculature by a physical therapist prior to and after surgery has a significant impact on the early recovery of urinary continence (68)
Capacity for organizational change	Regulations, rules, policies	Reduce urinary incontinence reliance on public health support (26)	
Social, political and legal factors	Payer or funder policies	It is causing costs if electrical stimulation and biofeedback with the help of an industrial device are used (31, 41) third-party payers then must bear the cost of biofeedback sessions (41) grant and financial support limitations, followed up sexual function last for only 12 months (71) and other economic (limited insurance) (52)	The components of the intervention were inexpensive (29) the use of biofeedback or electrical stimulation does not appear to be essential makes intervention less costly (30) low-cost physical therapy (23, 34, 35). Less-intense therapy may be more cost-effective (24)

## 3. Results

**Figure 1:**
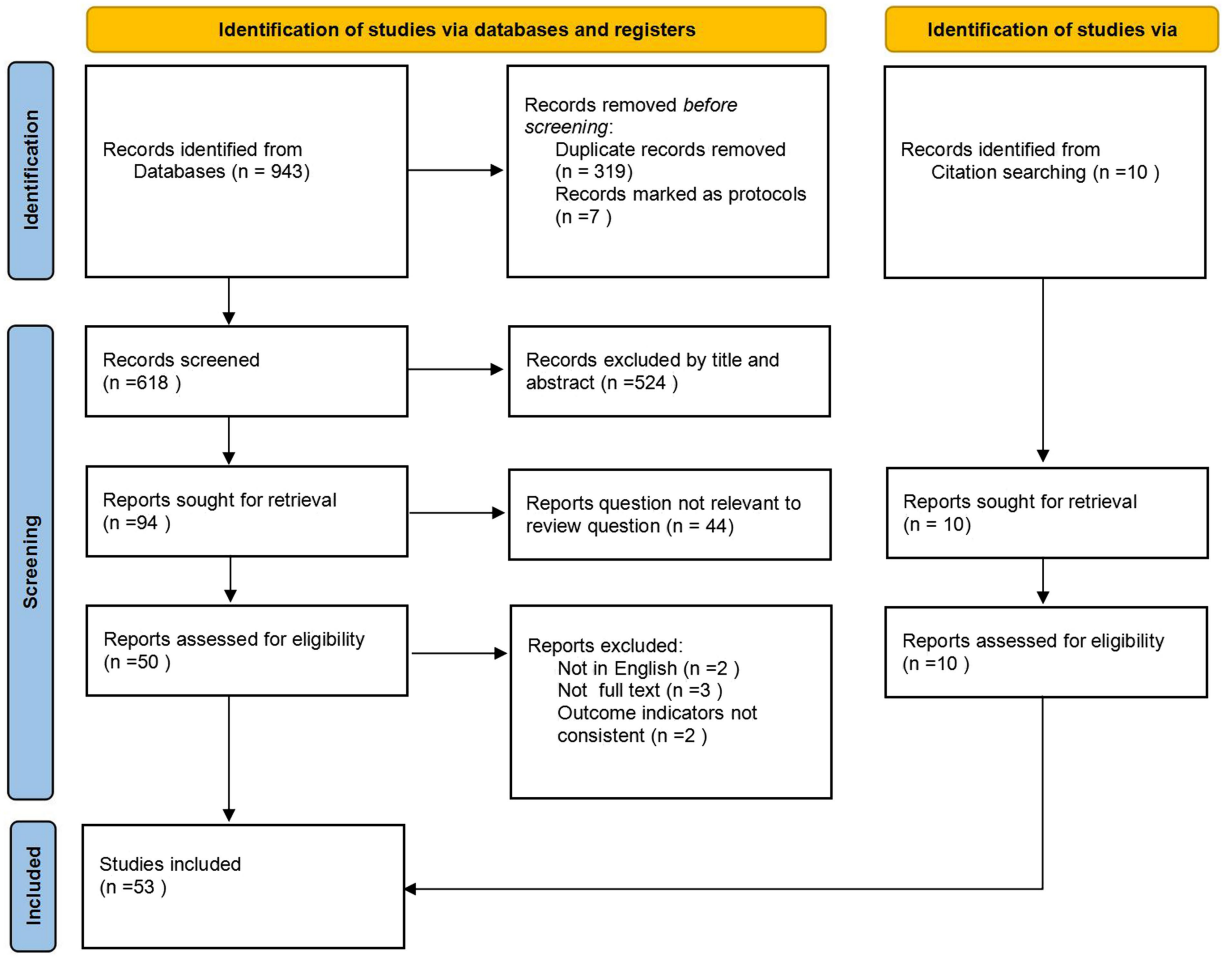
Illustrates the PRISMA flow diagram.

The search yielded 943 individual citations after removing 319 duplicate citations. Following the screening of titles and abstracts, 94 articles were selected for full-text review. Fifty-three met the inclusion criteria and are represented in this scoping review. See Figure 1 for the PRISMA flowchart.

Of these 53 studies, nine studies were from the United States, six from Brazil, six from Italy, four from Australia, three from the Netherlands, three from the United Kingdom, three from Canada, two from Germany, Denmark, Norway, Spain, Egypt, and South Korea, and one each from China, Taiwan, Poland, Japan, and Turkey. There were 41 randomized controlled trials, two cohort studies, and six articles that did not specify the study design. The three included studies were published by the same person, two studies were published by three others, and the two articles by Joanne were based on the same study. Therefore, data from these studies were counted only once in the total sample size calculation. In all 42 studies where age was reported, the mean age of all patients was 63.77 years. The cumulative number of participants was 5,521. The characteristics of the included studies are given in Table 2.

**Table 2:** Study characteristics.

N	First author	Country	Year	Study design	Age	Sample size	Purpose	Intervention time	Intervention
1	Hirschhorn	Australia	2013	Cohort-study	63	139	To assess the efficacy of a multicomponent, theory-based intervention in the provision/receipt of preoperative PFMT among men undergoing radical prostatectomy	/	Multicomponent, theory-based intervention
2	Santos	Brazil	2017	RCT	63.9	13	To analyze the application of physical therapy techniques (PFMT) in the recovery of urinary incontinence after prostatectomy.	After prostatectomy.	Intervention group: exercises and received biofeedback training control group: exercises alone
3	Goode	USA	2011	Prospective RCT	66.7	208	To determine whether the technologies of biofeedback and pelvic floor electrical stimulation enhance the effectiveness of behavioral therapy.	A year after radical prostatectomy	Behavior group: pelvic floor muscle training and bladder control strategies. Behavior plus group: dual channel electromyograph biofeedback and daily home pelvic floor electrical stimulation at 20 Hz, current up to 100 mA Control group: delayed treatment
4	Floratos	USA	2002	Prospective randomized trial	64	42	To evaluate the comparative effectiveness of electromyographic (EMG) biofeedback with verbal instructions as learning tools of pelvic muscle exercises (PMEs) in the early management of urinary incontinence after radical prostatectomy	After radical retropubic prostatectomy	Group A: 15 sessions of EMG biofeedback (three times weekly, 30 min each). Group B:verbal feedback
5	Jürgen	Germany	2005	Retrospective cohort survey		132	evaluated the clinical usefulness of early-onset pelvic floor reeducation (EPFR) as compared with later-onset pelvic floor reeducation (LPFR) in patients undergoing radical retro-pubic prostatectomy	A year after radical prostatectomy	Early-onset pelvic floor reeducation (EPFR) and later-onset pelvic floor reeducation (LPFR)
6	Tienforti	Italy	2011	RCT	65.5	32	To evaluate the efficacy of preoperative biofeedback (BFB) combined with an assisted low-intensity programme of postoperative perineal physio-kinesitherapy in reducing the incidence, duration and severity of urinary incontinence (UI) in patients undergoing radical prostatectomy (RP).	Perioperative + postoperative	Intervention group: training session with BFB, supervised oral and written instructions on Kegel exercises and a structured programme of postoperative exercises on the day before open RP. After RP, patients received control visits, including a session of BFB, at monthly intervals only. Control group: after catheter removal, only oral and written instructions on Kegel exercises to be performed at home.
7	Manassero	Italy	2007	RCT	67.35	152	To assessed the effects of early, intensive, prolonged pelvic floor exercises (PFE) on urinary incontinence following bladder neck (BN) sparing RRP	After radical prostatectomy	T group received instructions regarding an intensive program of PFE. The control (C) group did not receive instructions.
8	Karlsen	Denmark	2021	RCT	62.95	35	To compare the effect of early couple counseling and pelvic floor muscle training (PFMT) with usual care for sexual and urinary dysfunction after RP.	After radical prostatectomy	Control group: usual treatment and care intervention group: the ProCan intervention in addition to usual treatment and care.
9	Overgard	Norway	2008	RCT	61	85	To assess the effects of guided pelvic floor muscle training on continence status and perceived problems with urinary function after RP.	After radical prostatectomy	Group A: physiotherapist guided pelvic floor muscle training. Group B: pelvic floor muscle training
10	Nilssen	Norway	2012	RCT	61	80	To study the effect of postoperative physiotherapist-guided pelvic floor muscle training(PFMT) on health-related quality of life (HRQoL) parameters in patients treated with radical prostatectomy (RP)	After radical prostatectomy	Group A: physiotherapist-guided PFMT. Group B: trained on their own
11	Heydenreich	Germany	2019	RCT	64.1	184	To assess the effects of sensorimotor training with an oscillation rod compared with standard pelvic floor muscle training on reduction of incontinence level, recovery time and the HRQL.	After radical prostatectomy	IG: standard pelvic floor muscle exercises and oscillating rod therapy CG: standard pelvic floor muscle exercises and relaxation therapy
12	González	Spain	2020	RCT		60	To ascertain whether an early 3 month treatment with electrotherapy and biofeedback restores continence in urinary incontinence patients after radical prostatectomy (RP).	After radical prostatectomy	Treatment group: physiotherapy consisting of electrotherapy and biofeedback. Control group: no specific treatment Both groups received a guide to perform pelvic floor exercises at home
13	C Prota	Brazil	2012	RCT	63.2	52	To test the Early postoperative pelvic-floor biofeedback effect on erectile function in men undergoing radical prostatectomy	After radical prostatectomy	Treatment group: receiving PFBT once a week for 3 months and home exercises or a Control group: received verbal instructions to contract the pelvic floor.
14	Tantawy	Egypt	2018	RCT	63.95	61	To investigate the effect of whole-body vibration training on stress urinary incontinence after prostate cancer surgery.	After prostate cancer surgery	Group 1:pelvic floor muscle training and whole-body vibration training Group 2:performed pelvic floor muscle training alone
15	Bales	USA	2000	/	60.1	100	To determine whether preoperative biofeedback training improves urinary continence overall or the rate of return of continence in men undergoing radical prostatectomy	Preoperative	Biofeedback group: graded pelvic muscle exercise training with biofeedback 2 to 4 weeks before surgery. Control group: pelvic muscle exercises without biofeedback
16	Dijkstra-Eshuis	Netherlands	2013	RCT	63.7	122	This study reports the effects of preoperative pelvic floor muscle therapy (PFMT) on SUI and quality of life (QoL) in men undergoing LARP.	Preoperative	Intervention group: PFMT with biofeedback. Control group: standard care
17	M.T. Filocamo	Italy	2005	/	/	300	Investigate the effectiveness of early pelvic floor muscle training (PFMT) patients undergone radical retropubic prostatectomy (RRP)	After radical prostatectomy	Treated group: structured PFMT program. Control group: not formally instructed in PFMT
18	Yu	Taiwan	2012		65.75	62	o explore the prevalence of sexual dysfunction and to assess the efficacy of PFME in sexual dysfunction following RP	After radical prostatectomy	Experimental group: PFME as part of regular daily activities. Control group: was taught the exercise in the third month
19	Zhang	USA	2015	RCT	65.33	279	To examine whether an intervention combining pelvic floor muscle exercise (PFME) and symptom self-management improves urinary continence and quality of life in prostate cancer patients.	After radical prostatectomy	A:biofeedback PFME plus a support group (BF + SUPPORT). B: biofeedback PFME plus telephone contact (BF + PHONE) C: usual care (UC)
20	Gislano	Brazil	2019	RCT	66.4	31	To evaluate the effects of a perioperative pelvic floor muscle training (PFMT) program versus usual care on early recovery of urinary continence and erectile function after RP.	Perioperative	Group 1: received usual post-RP care. Group 2: Physical therapy received two pre-RP physical therapist-guided PFMT sessions, including exercises and electromyographic biofeedback
21	Cornel	Netherlands	2005	On-randomized study	64	57	To study the effect of early pelvic floor re-education on the degree and duration of incontinence and to evaluate the results of radical retropubic prostatectomy	After radical prostatectomy	Pelvic floor re-educating program
22	Pan	China	2019	Preexperimental single-group study	/	43	To examine the effects of using resistance band pelvic floor muscle exercise for patients after RAS prostatectomy.	After radical prostatectomy	Resistance band pelvic floor muscle exercise
23	Joanne	USA	2008	RCT	59.8	126	To examine the effects of systematic postoperative pelvic floor training (PFT) on LUTS intensity	Post-prostatectomy	Intervention group: an additional 4 weeks of PFT immediately following catheter removal Control group: brief instructions for exercising pelvic floor muscles before surgery and the offer of a biofeedback evaluation session 1 month following catheter removal
24	Zhang	USA	2017	RCT	64.8	267		After radical prostatectomy	(1) biofeedback PFME plus a support group (BF + Group), (2) biofeedback PFME plus telephone (BF + Phone), (3) usual care (UC)
25	Lúcia	Brazil	2010	Prospective RCT	/	73	Tested the effectiveness of biofeedback-pelvic floor muscle training in improving urinary incontinence in the 12 months following radical prostatectomy	After radical prostatectomy	Treatment group: biofeedback-pelvic floor muscle training once a week for 3 months. Control group: home exercises
26	Carla	Brazil	2018	RCT	57.93	123	To investigate the effect of electrical stimulation and pelvic floor muscle training on muscle strength, urinary incontinence and erectile function in men with prostate cancer treated by radical prostatectomy.	After radical prostatectomy	Control group patients were instructed to perform three types of home exercises to strengthen the pelvic floor and (G3, *n* = 42) electrical stimulation: patients in this group were also instructed to perform exercises as group G2, and also received anal electro-stimulation therapy, twice a week for 7 weeks.
27	Joanne	Australia	2020	RCT	62.85	97	We aimed to assess the impact of PFM training on ED and QoL in a prospective study	Before radical prostatectomy	Control group:3 sets/d PFMT Intervention group:6sets/d in standing, commencing 5 weeks before RP
28	Geraerts	Belgium	2015	RCT		33	To determine whether patients, minimum 12 months after RP, with persistent ED experienced a better recovery of ED with PFMT compared with patients without treatment. Our secondary aim was to investigate the effect of PFMT on climacturia.	After radical prostatectomy	Treatment group started PFMT immediately at 12 months post operation. Control group started at 15 months after RP. All patients received PFMT during 3 months.
29	Joanne	Australia	2019	RCT	63	97	Developed a novel PFM training program focussed on activating fast and slow twitch muscle fibres.	Five weeks prior to RP surgery; continued for 12 weeks post-RP	Group A: six sets of PFM exercises per day, with each set comprising 10 fast (1 s duration) and 10 slow (10 s duration) contractions with an equal rest time, providing a total of 120 contractions per day. Group B: three sets of PFM exercises per day, with 10 contractions per set, aiming to hold for a duration of 10s, with an equal rest time, providing a total of 30 contractions per day.
30	SIGRID TIBAEK	Denmark	2006	randomized, single-blind study	69	49	To evaluate the effect of preoperative pelvic floor muscle training (PFMT) in men scheduled for transurethral resection of the prostate (TURP)	Preoperative	Group A: an individual lesson (last 1 h), home exercises (PFM strength and endurance exercise) and three group treatments (isolated PFM contractions, strength exercises, endurance exercises and PFM contractions).
31	MARCHIORI	Italy	2010	RCT	/	332	Investigated if a post surgery tutored and personal trained pelvic floor re-educational program improves continence recovery more than pelvic floor exercises performed by patients on their own.	After surgery	Group A: intensive tutored pelvic training program. Group B: control group
32	Marianne	UK	2009	RCT	/	53	Test the effectiveness of instruction in self-care PME/biofeedback using a PME protocol to increase urethral resistance and to reduce the durations, amounts, and episodes of postprostatectomy urine losses.	After surgery	Group A: performed the exercise using a prescribed home PME protocol from week 3 to week 12 after surgery
33	Daniel	Canada	2018	RCT	/	86	Examined the feasibility and effects of prehabilitation on perioperative and postoperative outcomes in men undergoing radical prostatectomy.	Preoperative	Group A: individualized exercise program including pelvic floor muscle strengthening instructions and a healthy lifestyle guide; engage in 60 min of home-based, unsupervised, moderate-intensity exercise on 3–4 days per week. Group B: pelvic floor muscle strengthening instructions and healthy lifestyle guide only.
34	Antonia	Italy	2010	RCT	59	118	To determine the benefit of starting pelvic floor muscle exercise (PFME) 30 d before RP and of continuing PFME postoperatively for early recovery of continence	Preoperative	Group A: start PFME preoperatively and continue postoperatively. Group B: start PFME postoperatively alone
35	Manish	Australia	2013	/	61	284	To evaluate the effect of a physiotherapist-guided pelvic floor muscle training program, commenced preoperatively, on the severity and duration of urinary continence after radical retropubic prostatectomy.	Preoperative postoperative	Group A: physiotherapist-guided pelvic floor muscle training from 4 weeks preoperatively. Group B:verbal instruction on pelvic floor muscle exercise by the surgeon alone both groups received PG-PFMT while in hospital, and recommenced their PFME after IDC removal on day 7 postoperatively and continued until continence return
36	Sara	UK	2022	RCT	71.1	63	To evaluate the effectiveness of the symptom management after radiotherapy (SMaRT) group intervention to improve urinary symptoms in men with prostate cancer.	After curative radiotherapy or brachytherapy	Group A: a 10-week symptom-management intervention including group support, education, pelvic floor muscle exercises, or a care-as-usual group.
37	Katarzy	Poland	2021	RCT	62.8	37	To assess the impact of pelvic floor muscle training (PMFT) in the treatment of stress urinary incontinence (SUI) in men after they received radical	After RP	The EG received 24 individual sessions of physiotherapist-guided PFMT (twice a week over 3 months) 2 weeks following the surgery
38	Sung-Woo Park	Korea	2021	RCT	69.25	49	To examine the changes from a combined exercise intervention after radical prostatectomy (RP) in older adult patients with prostate cancer.	Postoperative week 3	Exercise group: received a combined exercise intervention (resistance, flexibility, and Kegel exercises) twice a week for 12 weeks, control group: received only Kegel exercises.
39	Gianna	Italy	2015	/	60	120	To compare the early vs. late use of pelvic floor electrical stimulation (FES) plus biofeedback (BF) in terms of time to recovery and rate of continence after radical prostatectomy (RP).	14 days after catheter removal (early program) ; 12 months after surgery(late program)	Group 1, the program started 14 days after catheter removal (early program) BF + FES program. Group 2, 12 months after surgery (late program) BF + FES program
40	Moore	Canada	2008	RCT	/	166	To test the effectiveness of weekly postoperative pelvic floor muscle training (PFMT) versus supportive telephone contact by a urology nurse for men at 4 weeks after radical prostatectomy.	4 weeks after radical prostatectomy	Treatment group: followed the standardized daily home routine and had weekly 30-min biofeedback-assisted PFMT for a maximum of 24 weeks.
41	Aylin	Turkey	2018	RCT	61.465	60	To determine the effect of pelvic floor muscle exercises (PFME/Kegel) training administered to patients scheduled for robot-assisted radical prostatectomy on postprocedural incontinence problems.	Perioperative	Treatment group: Pelvic floor muscle exercises control group: no exercise
42	Dubbelman	Netherlands	2010	RCT	64	66	To compare the effect on the recovery of incontinence after retropubic radical prostatectomy (RRP) of intensive physiotherapist-guided pelvic floor muscle exercises (PG-PFME) in addition to an information folder, with PFME explained to patients by an information folder only (F-PFME), and to determine independent predictors of failure to regain continence after RRP.	After retropubic radical prostatectomy	PG-PFME group: verbal instruction and an information folder on PFME received a maximum of nine sessions with the physiotherapist. F-PFME group: verbal instruction and an information folder on PFME with no further physiotherapist guidance
43	Serda	Spain	2013	RCT	71.435	66	To design and implement a rehabilitation program based on pelvic floor muscle training (PFMT) to improve the urinary incontinence (UI).	After treatment	Experimental group: The progressive strength program. Control group: watchful waiting
44	Ahmed	Egypt	2012	RCT	57.43	80	To assessed the effect of pelvic muscle exercises (PME), electrical stimulation (ES) and biofeedback (BFB) on UI after RP.	After Radical Prostatectomy	Group I: instructions about PME. Group II: received ES. Group III: received ES plus BFB
45	Glazener	UK	2011	RCT	62.35	411	To determine the Clinical effectiveness and cost-effectiveness of active conservative treatment, compared with standard management, in regaining urinary continence at 12 months in men with urinary incontinence at 6 weeks after a radical prostatectomy or a transurethral resection of the prostate (TURP).	After Radical Prostatectomy	Intervention group: Active conservative treatment [pelvic floor muscle training (PFMT) delivered by a specialist continence physiotherapist or a specialist continence nurse]. Control group: standard management
46	Kampen	Belgium	2000	RCT	65.47	102	To investigate whether there was any beneficial effect of pelvic-floor re-education for patients with urinary incontinence as a result of radical prostatectomy	After Radical Prostatectomy	Treatment group: pelvic-floor re-education programme. Control group: placebo therapy
47	Zhang	USA	2007	RCT	61.5	29	To examine the effect of combined pelvic floor muscle excise and a support group on postprostatectomy urinary incontinence and quality of life	After Radical Prostatectomy	Control group: practice PFME at home. Support group: attend six bioweekly group meetings facilitated by a health psychologists
48	Burgio	USA	2006	RCT	60.9	112	To tested the effectiveness of preoperative biofeedback assisted behavioral training for decreasing the duration and severity of incontinence, and improving quality of life in the 6 months following radical prostatectomy	Preoperative	Control group: usual care Intervention group ;biofeedback assisted behavioral training plus daily home exercise
49	Yamanishi	Japan	2010	RCT	66.6	56	To evaluated electrical stimulation combined with pelvic floor muscle training for urinary incontinence after radical prostatectomy in a randomized controlled study	After Radical Prostatectomy	Treatment group ;PMFT+ES. Sham group: PMFT
50	Sung-Woo Park	Korea	2012	RCT	69.25	49	To examine the changes from a combined exercise intervention after radical prostatectomy (RP) in older adult patients with prostate cancer	After radical prostatectomy	The exercise group: received a combined exercise intervention (resistance, flexibility, and Kegel exercises) twice a week for 12 weeks The control group: received only Kegel exercises.
51	Laurienzo	Brazil	2013	RCT	/	49	To evaluate electrical stimulation of the pelvic floor muscles prior to radical retropubic prostatectomy to accelerate the recovery of continence.	Preoperative	Control group: did not perform any intervention. Exercise group: Pelvic exercises Electrical stimulation: electrical stimulation and rectal pelvic exercises
52	Paekh	USA	2003	/	58.55	38	To determined whether preoperative and early postoperative biofeedback enhanced PFE with a dedicated physical therapist would improve the early return of urinary incontinence.	Preoperative and early postoperative	Control group: without formal PFE instructions Treatment group: physical therapy and underwent PFE sessions before and after surgery
53	Moore	Canada	1999	RCT	67	58	To access the effectiveness of intensive conservative treatment on and the impact of urinary incontinence after radical retropublic prostatectomy.	After Radical retropublic Prostatectomy	Group 1: standard treatment. Group 2: intensive PME. Group 3: PME + ES

### 3.1. Guideline factors

#### 3.1.1. Barriers

Inconsistent definition, measurement criteria, and treatment: Four studies mentioned inconsistent definition, measurement, and treatment (12, 23, 24, 42). Many of the mentioned trials lacked standardized outcome measures (23). The definition of UI varied in almost all studies. The means of measuring QoL were different, and the type of pad test was also different (20 min, 1 h, 24 h, number of pads, weight of pads, and number of men using pads, etc.). Only by aligning these definitions to the guidelines can the degree of improvement in incontinence following PFMT be measured more accurately.

Various influencing factors of the intervention effect: When establishing a clinical intervention program, the program’s accessibility and the appropriate duration of the intervention should be considered. Verbal instruction in PFMT for postoperative PCa patients is insufficient (30). The compatibility of the recommended behavior refers to how closely the recommended behavior matches the actual behavior. People with certain functional impairments who are unlikely to benefit from PTFM should also be excluded.

PFMT was found to be ineffective in improving UI in eight studies (31, 34, 40–45) and ED in four studies (34, 40, 43, 44); two other studies (45, 46) concluded that pelvic floor muscles were ineffective in improving patients’ QoL (see Table 1).

#### 3.1.2. Facilitators

##### 3.1.2.1. Simple and practical intervention

PFMT has significant advantages in terms of feasibility, including the simplicity of pelvic floor exercises (29), the non-invasive nature of the physiotherapy approach (33), and the fact that some studies indicate that the use of biofeedback or ES does not appear to matter, making the intervention more practical (30).

##### 3.1.2.2. Obvious benefits of intervention

Thirty-five studies concluded that pelvic floor exercises are effective in improving UI in patients, three studies found that PFMT improves ED, and six studies found that PFMT improves QoL in postoperative patients. Studies have shown that exercise improves anxiety and depression in patients. Prior to surgery, PFMT of greater duration, frequency, or intensity is more likely to be beneficial (see Table 1).

### 3.2. Individual health professional factors

#### 3.2.1. Barriers

Limited healthcare professionals’ knowledge and skills: A lack of knowledge or expertise among target healthcare professionals, especially urologists, about the target condition affects the conduct of clinical PFMT (29). The extent to which the target healthcare professionals understand and are familiar with the recommendations affects the conduct of PFMT, such as the intensity of the program and the position in which pelvic floor muscles contract. Interventions may be biased due to the surgeon’s learning curve.

Targeted healthcare professionals can self-monitor or provide feedback to enhance compliance with recommendations. The main problems presented here include issues with the experimental setup during PFMT, the relatively small number of patients (34, 35, 38, 42, 47, 58, 59, 61), and the short follow-up period, among others.

Lack of optimal individual treatment: Healthcare professionals can plan the necessary changes to which they can adhere to. However, this can be a hindrance when healthcare professionals conduct PFMT with problems in the setting of the experiment, such as the failure to establish an optimal individual exercise regimen.

#### 3.2.2. Facilitators

Experienced physicians and nurses: The support of physicians and nurses experienced in voiding disorders can shorten the speed and time to achieve voiding (26). Surgeons with a thorough understanding of instructions and techniques can greatly assist patients in adequately performing PFMT.

The facilitating factors were analyzed using a rigorous study protocol with all randomized patients, and an evaluation panel that was blinded and provided the intervention over as long a period as possible was shown to be more effective. Randomized controlled trials should be well-designed, involving multicenter trials with adequate sample sizes; they should utilize validated outcome measures and implement long-term follow-up procedures (67). Sophisticated experimental designs will result in better PFMT exercise.

### 3.3. Patient factors, patient motivation, and patient behavior

#### 3.3.1. Barriers

PFMT results may depend on patients’ motivation (31). Failure to ensure patient compliance and adherence to exercise leads to uncertainty in the effectiveness of exercise (49, 71). Four studies highlighted fatigue, lack of transportation and time, and long distance to study sites as reasons why patients at home declined to exercise consistently (36, 52, 56, 67). Physical exercise requires better compliance and persistence and should be performed regularly.

#### 3.3.2. Facilitators

Patients take charge of their urinary health, and self-care activities contribute to improving compliance and adherence to exercise (32, 72). Moreover, intensive and supervised programs have produced better results than self-training programs (66).

### 3.4. Incentive and resources

#### 3.4.1. Barriers

Three studies reported that the lack of professional healthcare, continuing care, and family burden hindered PFMT implementation (38, 55, 64). For instance, a study reported that no local public provider was called upon to provide preoperative PFMT over the post-intervention period (29). Furthermore, a range of factors related to age, gender, social, and demographic background may prevent the accessibility and availability of assistance and the lack of professional healthcare support for older adults in need (64).

#### 3.4.2. Facilitators

The characteristics of PFMT exercise make it less dependent on technology and hospital facilities (32). A continuing education system is crucial to facilitate participation and adherence to treatment recommendations. The use of a low-intensity, supervised program will be more cost-effective for peripheral urological clinics.

### 3.5. Professional interactions

#### 3.5.1. Barriers

Team processes of PFMT as a teamwork operation: The results depend considerably on the unity and cooperation among the members. Some studies suggest frequent collaboration with a physiatrist (31), while others argue that it is unnecessary (59). Physical therapy is dominated by physical therapists, so the results are often related to the dedication of physical therapists (31). Intensive instruction from a physical therapist is often time-consuming and expensive, which seems unnecessary (59). As the professionals closest to the patients, nurses who lack knowledge about or experience in biofeedback equipment techniques should be trained (41).

#### 3.5.2. Facilitators

Physiotherapists who educate patients about pelvic floor muscles before and after surgery have a significant impact on early recovery from incontinence (64). Close monitoring by physiotherapists and physiotherapists’ interests are significant factors in the effectiveness of PFMT (39). Moreover, effective interaction between patients and healthcare professionals contributes to the effectiveness of PFMT (51).

### 3.6. Capacity for organizational change

Regulations, rules, and policy barriers: Some studies believe that the public health sector has limited support for pelvic floor exercise (26). Therefore, healthcare personnel should reduce UI reliance on public health support.

### 3.7. Social, political, and legal factors

#### 3.7.1. Barriers

The payer or funder policy may affect the implementation of necessary changes if using ES and biofeedback. Additional expenses may be incurred because of specialized equipment (31, 41).

Third-party payers must bear the cost of biofeedback sessions (41). Funding and financial support are limited by a follow-up of sexual function lasting only 12 months (71) and other economic factors (limited insurance) (52).

#### 3.7.2. Facilitators

Regardless of biofeedback or ES, pelvic floor exercises and psychotherapy are inexpensive, and even less-intense interventions can be effective (23, 24, 29, 30, 34, 35).

## 4. Discussion

This scoping review aimed to map the literature on the implementation of PFMT in PCa and provide an opportunity to identify research gaps, types, and sources of evidence to guide clinical practice. It highlights the barriers and facilitators affecting PFMT implementation in clinical practice. The findings of this review show that men diagnosed with PCa can encounter personal and social enhancements on the one hand, but on the other hand, they also need to face some obstacles in the process of participation. These barriers and facilitators mainly originate from four aspects: the design of research schemes, healthcare professionals, patient factors, and policy or social support. Many of the factors identified as barriers were also facilitators—healthcare professionals with extensive experience may be helpful for study development and for improving patient adherence (27), but healthcare professionals can also hinder patient adherence when their knowledge and skills are limited (64). This scoping review also identified that men PCa patients face similar obstacles to other older adult cancer patients when participating in PFMT exercises, such as cancer treatment side effects, age-related functional decline, a lack of time, and long distances from medical institutions (52, 67). A multidisciplinary team (MDT) including physiotherapists, nurses, and clinical scientists is essential for effective PFMT implementation and coordination. It has been reported that a group setting may increase patients’ motivation to maintain PFME and improve their enthusiasm for participation (61).

### 4.1. Unified standards

Most trials included in this review followed different protocols in terms of intervention type, duration, and intensity. A large and powerful test using the general scheme and the general standardized outcome measurement is required to determine the effect of the PFMT of a specific scheme. A replication study using similar protocols in different populations can also help determine the populations that may benefit from specific conservative management methods (12). The definition and measurement of outcomes differed between the included tests. Future trials should use widely accepted and validated outcome indicators, such as those of the International Continence Society (ICS) (25). The main outcome indicator should be the self-reported user interface of participants or its impact on their QoL (74). Other objective measurement methods, such as the urine pad test or a urine diary, can determine whether urine control has been achieved. Details on the implementation of specific PFMT and the measurement criteria should be considered when formulating the guidelines. In summary, carefully designed clinical trials are needed to clarify the role of PFMT.

### 4.2. Guidance from healthcare professionals

Implementing a recommendation based on knowledge and skill may require targeted efforts to disseminate that new knowledge or skill (20). The targeted healthcare professionals may relapse to previous behaviors, forget to adhere, lack the necessary support or resources to maintain adherence, or lack time or skills to plan necessary changes. Therefore, before developing an intervention program for implementation, it is essential to gather routinely collected data on human resources for health, conduct interviews, or hold focus group discussions with targeted healthcare professionals.

### 4.3. Personalized exercise program

According to the literature, the exercise prescription recommended by tumor team members (such as doctors or nurses) can help improve cancer patients’ exercise compliance (75, 76). In clinical practice, however, doctors and nurses rarely guide patients through detailed PFMT, often limiting their instructions to oral guidance only. From a research standpoint, given the specific needs of PCa patients, we should emphasize the adjustment and adaptation of personalized preferences and needs. Additionally, considering the challenges and obstacles related to the side effects of treatment and functional decline, further research is needed to examine the factors related to the persistence of exercise in this population and to explore more feasible and convenient operation sites and methods. Furthermore, it is necessary to broaden the scope, including investigating group-based, supervised, and remote online exercise programs at home and to remove barriers for PFMT implementation related to acceptability, preferences, and potential obstacles.

### 4.4. Assistance with equipment

This study found that the patient transfer and communication process between different levels of care—between health and social services and between target healthcare professionals and target patients—is critical for successfully implementing PFMT. PFMT intervention strategies are frequently resource-intensive, necessitating personnel, equipment, and clinic space (76). When performing contraction exercises, patients frequently struggle to determine whether the contraction mode is correct. Consequently, some equipment and instruments are critical for patients following PCa surgery (41). Teamwork is essential for the correct and standardized implementation of PFMT, particularly physical rehabilitation assistance. Some studies believe that the efficacy of PFMT depends on interactions with healthcare professionals (51) because exercise in interaction and motivation improves enthusiasm. Regarding regulations, rules, policies, and payers or funders, the rehabilitation costs of PFMT should be considered in the scope of medical insurance as soon as possible. Many hospitals and communities have struggled to implement PFMT due to the lack of specialized equipment for measuring ES, biofeedback, and patient muscle contraction strength.

### 4.5. Patient’s mastery of their condition

Studies have confirmed the importance of patients’ motivation to participate in the exercise process (31). In a randomized controlled clinical trial, patients with persistent UI and PCa implemented PFME practice and symptom self-management under peer support. The results showed that the symptoms and incontinence problems in the intervention group were less severe than those in the control group. This patient-centered approach reduces the dependence on technology and hospital facilities and enables patients to be responsible for their urinary health (32). Furthermore, the ability or perceived ability of targeted healthcare professionals to motivate patients to adhere is also critical.

In summary, PFMT is still in the initial stage of application in domestic patients with PCa after surgery and faces many obstacles in the promotion process. It is necessary for medical staff and health management departments to overcome the obstacles together and promote its clinical application in PCa to improve postoperative UI and patients’ QoL.

This scoping review adds to the existing literature and highlights new findings, closing the knowledge–practice gap. Future research on overcoming barriers and maximizing facilitators is needed to improve, modify, or complement existing evidence on PFMT implementation practices.

## 5. Limitations

First, although we conducted a thorough search using broad selection criteria, we might have missed some published papers in this field. We could not screen titles and abstracts independently due to resource constraints, which might have affected the included research. Second, not all the studies included in this article were subjected to rigorous quality control, and there is the possibility of an uneven hierarchy in the study settings. Third, although two researchers summarized all the factors that promoted or hindered PFMT implementation, some personal subjectivity might have affected the results when summarizing descriptive information.

## 6. Conclusion

Our study identified multiple barriers to and facilitators of PFMEs in PCa patients in a practical environment. This study is an important step toward rationally designing intervention measures. To promote PFMEs among PCa patients, targeted public health intervention measures should use social relations and social support to exercise pelvic floor muscles in PCa patients after surgery.

## Data availability statement

The original contributions presented in the study are included in the article/supplementary material, further inquiries can be directed to the corresponding author.

## Author contributions

LW wrote the manuscript, analysis and interpretation of the data. YL drafting the article or revising it critically for important intellectual content. ZQ helped final approval of the version to be submitted. WW formed the conception and design of the study. All authors contributed to the article and approved the submitted version.

## Conflict of interest

The authors declare that the research was conducted in the absence of any commercial or financial relationships that could be construed as a potential conflict of interest.

## Publisher’s note

All claims expressed in this article are solely those of the authors and do not necessarily represent those of their affiliated organizations, or those of the publisher, the editors and the reviewers. Any product that may be evaluated in this article, or claim that may be made by its manufacturer, is not guaranteed or endorsed by the publisher.
